# Analysis of Genetic Variation of Two NADC30-like Strains of Porcine Reproductive and Respiratory Syndrome Virus in China

**DOI:** 10.2174/1874357901711010090

**Published:** 2017-06-30

**Authors:** Zhi Zhou, Jiajun Wu, Shuo Zhang, Bo Hou, Tao Han, Jing Wang, Qi Xu, Dongyue Wang, Yinyi Liu, Shengpeng Xin, Xinyan Zhai

**Affiliations:** China Animal Disease Control Center, OIE Porcine Reproductive and Respiratory Syndrome Reference Laboratory, No. 20 Maizidian Street, Chaoyang District, Beijing 100125, China.

**Keywords:** PPRSV, NADC30-like strain, Genetic diversity, Virus

## Abstract

**Background:**

NADC30-like strains of porcine reproductive and respiratory syndrome virus first appeared in Chinese swine herds in 2012.

**Objective and Method:**

To explore the possible genetic diversity of these strains, we sequenced and analyzed the complete genomes of two NADC30-like strains. These isolates shared 95.4% homology with NADC30.

**Result:**

The two strains displayed a discontinuous deletion of 131 amino acids in NSP2, mutations of amino acids in GP3 and GP5, and a 3-nucleotide deletion in the 3′ untranslated region. Phylogenetic analysis showed that the two isolates formed a new branch and clustered in a subgroup with NADC30 isolates from North America.

**Conclusion:**

We conclude that the above two NADC30-like strains may have been introduced from North America to China, where they acquired new genetic diversity.

## INTRODUCTION

Porcine reproductive and respiratory syndrome (PRRS) is one of the most important infectious diseases in swine. This disease leads to reproductive failure in sows and respiratory disease in pigs of all ages [1, 2]. PRRS virus (PRRSV), which belongs to the *Arteriviridae* family in the order *Nidovirales*, is an enveloped virus with a single-stranded positive-sense RNA genome [1-3]. The whole PRRSV genome is ~15 kb and contains at least 10 overlapping open reading frames (ORFs): ORF1a and ORF1b encode viral nonstructural proteins, whereas ORF2a, ORF2b, and ORF3–7 encode viral structural proteins [4-7].

PRRSV is divided into European (Type 1) and North American (Type 2) genotypes, which share only 60% homology at the nucleotide level [7, 8]. Although PRRSV isolates are always identified in their respective geographic regions, coexistence of the two types has been found in Europe, North America, and Asia. Such coexistence has had a major impact on PRRS diagnosis and management [9-11]. In China, emergence of PRRSV Types 1 and 2 has been reported previously [12, 13], although the most prevalent isolates in China are Type 2 strains.

Since 2012, NADC30-like PRRSV has been reported in Chinese swine herds and has caused huge economic losses [14]. To explore the epidemic status and genetic diversity of NADC30-like PRRSV in China, we isolated two NADC30-like strains, sequenced their complete genomes, and compared their genetic relationships with previously examined NADC30 and NADC30-like PRRSV strains. We analyzed gene sequences of the 5′-untranslated region (UTR), 3′-UTR, hypervariable nonstructural protein 2 (Nsp2) gene, and most of the major viral structural proteins encoded by ORF3 and ORF5 of the two PRRSV isolates.

## MATERIALS AND METHODS

### Ethics Statement

This study was carried out at private farms. Animals were sampled with the permission of the farm’s managers. The animal field trial in this study was approved by the Animal Care and Ethics Committee of China Animal Disease Control Center. Conventional animal welfare regulations and standards were taken into account.

### Virus Isolation

Twenty-six clinical samples of lungs, lymph nodes, livers, kidneys, and sera were collected from different farms in China. Samples were screened by a conventional RT-PCR assay for both genotypes of PRRSV [13]. Samples that tested positive by PCR were further tested by real-time RT-PCR for differentiation of PRRSV Types 1 and 2 [13, 15]. Positive tissues were homogenized with DMEM, frozen/thawed three times, and centrifuged at 10,000 × *g* for 15 min. Supernatant was passed through a 0.22-μm filter and inoculated on primary porcine alveolar macrophages (PAMs) and Marc-145 cells for virus isolation, as previously described [16, 17]. Inoculated cells were maintained at 37 °C in a 5% CO_2_ atmosphere and monitored daily for cytopathic effects.

### RNA Extraction and Genome Sequencing

Total RNA was extracted from cell cultures by using an RNeasy Mini kit (Qiagen, Germany) according to the manufacturer’s instructions. Whole genomes were sequenced as described previously [18]. Briefly, 16 overlapping fragments covering the whole viral genome were amplified by RT-PCR using the corresponding primers. The PCR products were purified with the E.Z.N.A. Gel Extraction Kit (OMEGA, USA) and cloned into pEASY-T1 (Transgen, China). Recombinant plasmids were sequenced with an ABI Automatic DNA Sequencer (Invitrogen, China) and spliced artificially. Gene fragments were sequenced in triplicate and assembled with ContigExpress in Vector NTI Advance 11 [19].

### Multiple Sequence Alignment and Phylogenetic Analysis

Multiple alignments were performed for each ORF separately using the represented available whole genome sequences for Type 2 PRRSV, including VR-2332, CH-1a, JXA1, MN184A, NADC30, HENAN-XINX, JI580, HEN-HB, and CHsx1401. Using the MEGA4.0 program [20], phylogenetic trees were constructed from aligned nucleotide sequences by the neighbor-joining method. Trees were subjected to bootstrap analysis with 1,000 replicates to determine the reliability percentages at each internal node of the tree. Whole genome sequences of all PRRSVs mentioned in this study are available in GenBank (Table **1**).

## RESULTS

### Virus Isolation and Complete Genome Sequence Analysis of Two NADC30-like Isolates

Following sample inoculation on primary PAM and Marc-145 cells for 3 days, cytopathic effects were observed on PAMs but not on Marc-145 cells for two inoculates and on both cell types for four inoculates. These six PRRSV inoculates were sequenced. Two isolates, NVDC-ZJ and NVDC-JS, exhibited higher homology with NADC30 than other PRRSV Type 2 isolates and were included in subsequent analyses. Excluding the poly (A) tails, complete genomes of NVDC-ZJ and NVDC-JS were 15,016 nucleotides in length. These two isolates shared 99.9% homology with each other, 95.4% homology with NADC30, 85.3% homology with VR2332 (representative Type 2 PRRSV), and 83.8% homology with JXA1 (representative highly pathogenic PRRSV).

Next, we analyzed the genomic characteristics of NVDC-ZJ in detail. Compared to other NADC30-like representative isolates, including HENAN-XINX, JL580, HEN-HB, CHsx1401 MN184A, and NADC30, the 5′-UTR and 3′-UTR of NVDC-ZJ had nucleotide homologies of 95.3–100% and 95.4–100%, respectively. Predicted nonstructural proteins encoded by ORF1a and ORF1b of NVDC-ZJ shared nucleotide homologies of 83.7–99.9% and 89.9–99.9%, respectively. Among all nonstructural proteins, the most variable protein within ORF1a, Nsp2, of NVDC-ZJ exhibited an amino acid identify of 67.0–99.9% with other isolates. Detailed identities for each coding region of NCDC-ZJ with other NADC30-like strains and representative North American isolates are summarized in (Table **1**).

### Sequencing Analysis of 5’-UTR and 3’-UTR of Two NADC30-like Isolates

The 5′-UTR and 3′-UTR are documented to be important regulatory elements in the PRRSV genome [7]. In this study, we compared the 5′-UTR and 3′-UTR nucleotide sequences of these isolates and other PRRSV isolates. The 5′-UTRs of the two isolates were 190 nucleotides in length, with no new deletions or insertions in this region. However, the 3′-UTRs were 148 nucleotides in length and contained a 3-nucleotide deletion at position 118–120 Fig. (**1**).

### Amino Acid Variation in Nsp2 of the Two NADC30-like Isolates

The most important and variable nonstructural protein of PRRSV, Nsp2 exhibited the lowest amino acid identity among the viral proteins. Compared to the classical PRRSV and HP-PRRSV, these two isolates exhibited discontinuous deletion of 131 amino acids within the Nsp2 protein, including an 111-aa deletion at position 322–432, 1-aa deletion at position 483, and 19-aa deletion at position 504–522 Fig. (**2**), which are similar to other NADC30-like strains. These discontinuous deletions were also observed in NADC30 and MN184 isolated in the United States [14].

### Amino Acid Alteration in GP3 of the Two NADC30-like Isolates

GP3 is a minor structural protein that is important for assembly of infectious PRRSV. GP3 is highly antigenic and has two fewer conserved epitopes at amino acids 67–74 (67YEPGRSLW74) and 74–85 (74WCRIGHDRCGED85) [21]. To understand the variations of the two epitopes, we studied alignment of the amino acid sequences of the two isolates in this study. We identified a 1-aa substitution at location 67 (Y67→F67) in the minimal epitope 67–74 and a 3-aa substitution at positions 83 and 85 (Y83→H83, G83→E83, D85→S85) in epitope 74–85 Fig. (**3**).

### Amino Acid Variation in GP5 of the Two NADC30-like Isolates

We further explored the genetic divergence in the two NADC30-like isolates of PRRSV. GP5, the most variable gene of PRRSV, was analyzed with other representative PRRSV Type 2 isolates. The GP5 amino acid sequence for each strain consisted of 200 residues with no deletions or insertions. Strains shared amino acid identities of 100% with each other, 92% with NADC30, 88% with MN184A, 95.4–96% with other NADC30-like strains, 85.5% with the representative HP-PRRSV JXA1 (isolated after 2006 in China), and 85.5% with VR-2332.

GP5 contains a signal peptide, three transmembrane proteins, an extravirion region, and an intravirion region [22]. The hypervariable region was observed in the signal peptide, extravirion region, and intravirion region. Compared to VR-2332, the signal peptide had four concurrent mutations at positions 3, 8, 14, and 16 (E3→G8, A8→V8, L14 →S14, and S16→F16) Fig. (**4**). We analyzed some known functional domains in the extravirion region of GP5, such as the primary neutralizing epitope B, decoy epitope A, and potential glycosylation sites. Decoy epitope A, comprised of A27/V27LVN near the primary neutralizing epitope, may delude most antibodies against GP5 and delay production of viral neutralizing antibodies [23]. We did not find any mutation of amino acids of epitope A. Primary neutralizing epitope B comprised amino acids at residues 37 to 44 (SHLQLIYN). This epitope is conserved and recognized by neutralizing monoclonal antibodies. No amino acid mutations of epitope B were found in either isolate Fig. (**4**).

There were at least three N-glycosylation sites (N-X-S/T) located in the extravirion region of GP5. The first potential N-glycosylation site was located in the hypervariable region (N32 or N33 or N34) of GP5 extravirion. The second and third potential N-glycosylation sites were located in residues N44 and N51. There were two concurrent mutations at positions 32 and 34 (S32→N32, D34→N34) within the two isolates Fig. (**4**). In the intravirion domain, there were nine concurrent amino acid mutations at positions 137(A→S), 151(R→K), 164(R→G), 168(E→D), 170(E→G), 185(V→A), 189(I→V), 191(R→K), and 192(V→I) Fig. (**4**).

### Phylogenetic Analyses of The Two NADC30-like Isolates with Other PRRSVs

Phylogenetic analysis was performed on the full-length genomic sequences of 17 PRRSV strains, including 14 Type 2 and one Type 1 strain from the GenBank database Table **1** and the two NADC30-like isolates in this study. In the phylogenetic tree, the two NADC30-like isolates formed a new branch within PRRSV Type 2 Subtype 2. They were closely related to CHsx1401 and NADC30 Fig. (**5**).

## DISCUSSION

PPRS is one of the most important infectious diseases in swine, causing reproductive failure in sows and boars and respiratory disease in pigs of all ages. North American PRRSV strains were first reported in China in 1996, followed by isolation of some epidemic strains in Chinese swine herds [12]. In 2006, unparalleled outbreaks of HP-PRRSV led to huge economic losses of pig husbandry and an extensive pandemic in China [24]. NADC30-like isolates were first reported in Henan province in 2012 and then isolated from other provinces in China [14, 25, 26].

To explore the genetic divergence of NADC30-like strains in Chinese swine herds, we investigated the complete nucleotide sequences of two isolates and analyzed their phylogenetic relationships with other PRRSV strains. Nucleotide sequences of the two isolates were highly homologous with sequences of other NADC30-like strains, and highly variable regions existed in NSP1β, NSP2, GP3, and GP5. Phylogenetic analysis divided the 16 PRRSV Type 2 strains into two subtypes: subtype 1 contained classical PRRSV and HP-PRRSV, and subtype 2 contained MN184 PRRSV and other NADC30-like isolates (including the two studied here). Moreover, the strains were closely related to and may have evolved from NADC30 by gradual variation and accumulation of genomic changes.

The Nsp2 gene is highly variable and includes naturally occurring mutations, insertions, and deletions, which might be the most important marker for monitoring genetic variation and evolution of PRRSV [27]. In 2006, an atypical PRRS outbreak in China was caused by a highly pathogenic PRRSV strain that contained a unique discontinuous deletion of 30 amino acids in the Nsp2 hypervariable region [24]. Our two studied isolates had a discontinuous deletion of 131 amino acids in the Nsp2 region, but the deletion differed from those of the HP-PRRSV strains and were more similar to those of the NADC30 strains. Some NADC30-like strains have HP-PRRSV virulence [14, 26]. Pathogenic analysis indicated the two isolates in this study were milder in virulence than the HP-PRRSV strains (data not show). Therefore, we conclude that the 131-aa deletion was not related to the virulence of PRRSV in China. Other research has associated PRRSV virulence with multiple factors. This issue is a complex question that will require further study.

GP3 and GP5 are important targets for analyzing the virulence and genetic variation of PRRSV, which is involved in viral immune evasion and minimization of the virus-neutralizing antibody response [28-30]. Compared to VR-2332, two epitopes of GP3 had four substitutions. Substitutions were observed in the signal peptide, extravirion, intravirion, and transmembrane regions. We also observed two potential N-glycosylation sites on GP5 in the two isolates. However, it is unknown whether these mutations or glycosylation sites are related to the virulence or immunogenicity changes of PRRSV. Nevertheless, our data indicate that the genetic diversity of PRRSV should be considered as a serious issue for PRRSV control and prevention.

## CONCLUSION

We have detailed the genomic sequences of two NADC30-like strains of PRRSV and explored their phylogenetic relationships with other PRRSV strains. The two NADC30-like isolates of PRRSV were introduced to mainland China from North America, thereafter gradually accumulating genomic changes.

## Figures and Tables

**Fig. (1):**
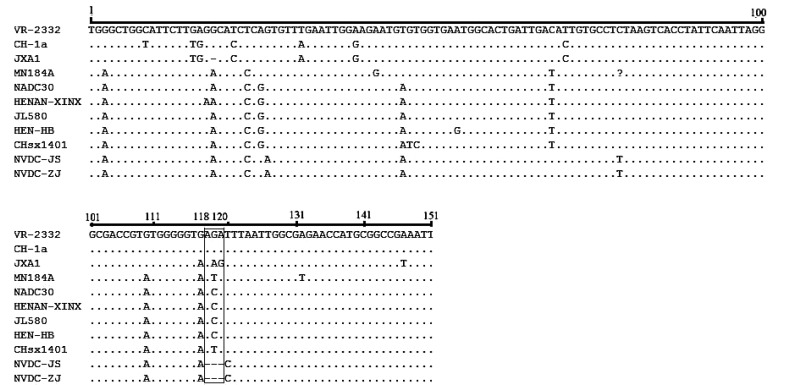
**Alignment of 3’-UTR sequences of two NADC30-like PRRSVs.** Deleted region is indicated by the black box.

**Fig. (2):**
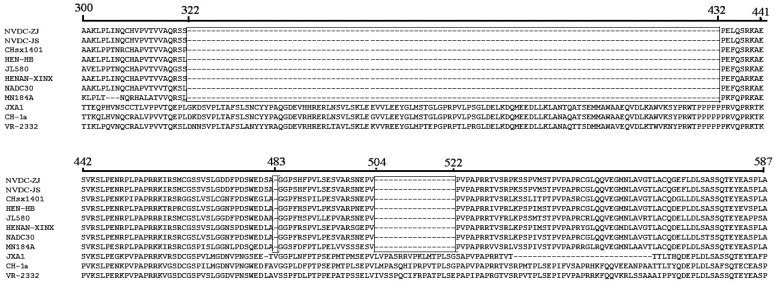
**Amino acid sequence alignments of the partial Nsp2 gene of two NADC30-like isolates.** Discontinuous deletion of 131 amino acids was found in the Nsp2 protein: 111-aa deletion at position 322–432, 1-aa deletion at position 483, and 19-aa deletion at position 504–522. All amino acid deletions are marked with black boxes.

**Fig. (3):**
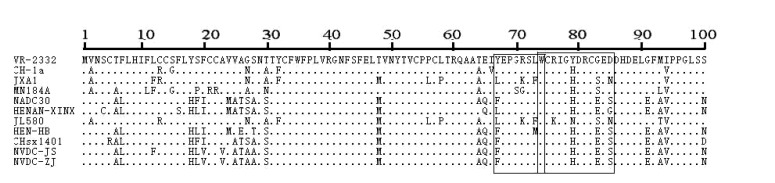
**Amino acid sequence alignments of the partial GP3 of two NADC30-like isolates.** The two NADC30-like isolates had two epitopes (black boxes) in GP3, located at position 67–74 (67YEPGRSLW74) and position 74–85 (74WCRIGHDRCGED85).

**Fig. (4):**
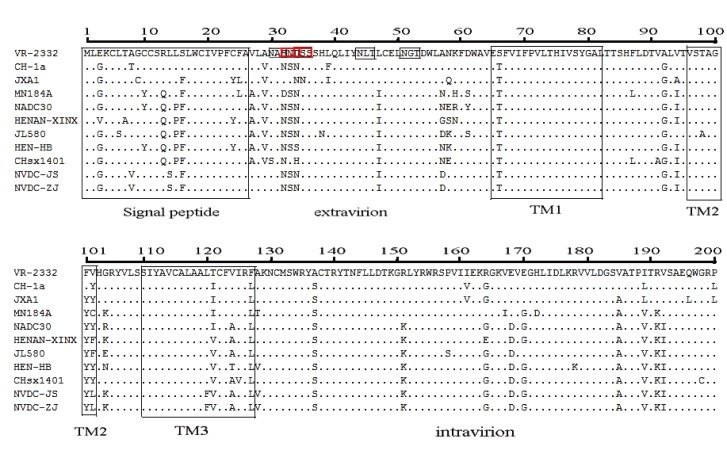
**Analysis of amino acid mutations in GP5 of the two NADC30-like isolates.** Functional domains (black boxes) were reported according to Han et al. (2006). Extra-and intra-virion epitopes of the isolates are boxed in the consensus sequence. Two concurrent mutations (red boxes) were found at positions 32 (S32→N32) and 34 (D34→N34). Other mutations are described in the text. TM: transmembrane region.

**Fig. (5):**
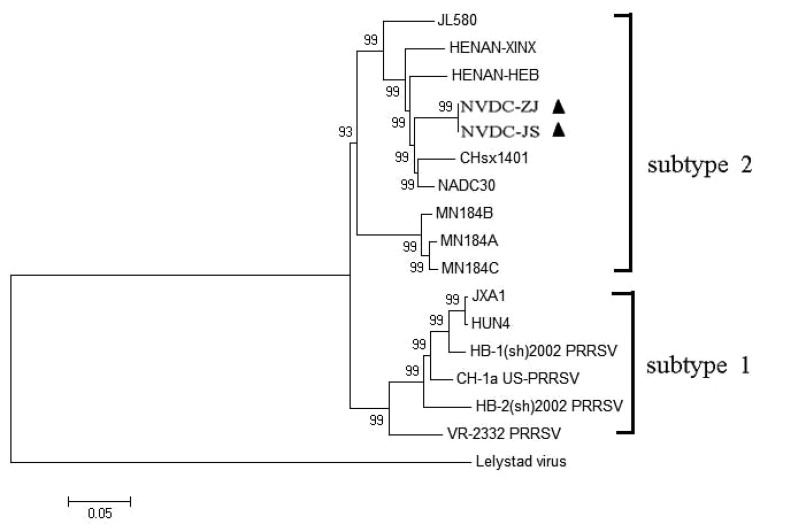
**Phylogenetic tree of two NADC30-like strains and other representative viruses.** Tree was constructed from aligned complete genomic sequences by the neighbor-joining method with the Kimura 2-parameter algorithm in MEGA4.1. Reliability was assessed by bootstrap analysis with 1000 replications. The two isolates of this study are indicated by dark triangle symbols.

**Table 1:** Amino acid analysis of nonstructural and structural proteins of NVDC-ZJ with other representative PRRSV strains

**ORF**	**Cleavage product**	**NVDC-JS**	**CHsx1401**	**HEN-HB**	**JL580**	**HENAN-XINX**	**NADC30**	**MN184A**	**JXA1**	**CH-1a**	**VR-2332**
**Nonstructural protein**											
**1a**	**Nsp1α**	**100**	**93.3**	**95.1**	**96.3**	**96.9**	**96.3**	**93.2**	**93.3**	**93.3**	**93.3**
	**Nsp1β**	**100**	**86.4**	**89.1**	**90.5**	**86.8**	**90.5**	**80.3**	**74.5**	**72.7**	**76.8**
	**Nsp2**	**99.9**	**85.5**	**82.4**	**79.7**	**89.2**	**90.3**	**73.4**	**67**	**67.1**	**69.3**
	**Nsp3**	**100**	**96.6**	**96.9**	**93.7**	**93**	**98.2**	**91.5**	**91.3**	**91.9**	**92.6**
	**Nsp4**	**100**	**99**	**97.1**	**92.6**	**94.1**	**97.5**	**92.6**	**93.6**	**93.1**	**94.1**
	**Nsp5**	**100**	**93.5**	**94.7**	**90**	**92.9**	**96.5**	**82.6**	**92.4**	**91.8**	**90**
	**Nsp6**	**100**	**95.8**	**100**	**95.8**	**100**	**100**	**100**	**95.8**	**95.8**	**100**
	**Nsp 7**	**100**	**94.6**	**95.4**	**90.7**	**95**	**96.1**	**88.8**	**84.6**	**85.7**	**89.2**
	**Nsp8**	**100**	**93.3**	**93.3**	**95.6**	**93.3**	**95.6**	**93.3**	**91.1**	**91.1**	**91.1**
**1b**	**Nsp9**	**100**	**96.2**	**95.3**	**96.1**	**96.2**	**98.7**	**96.9**	**95.8**	**95.9**	**97.7**
	**Nsp10**	**100**	**98.2**	**98**	**97.7**	**97.7**	**98.2**	**93.6**	**93.4**	**93.4**	**94.8**
	**Nsp11**	**100**	**94.2**	**98.7**	**97.8**	**98.2**	**99.1**	**92.4**	**96.4**	**96**	**93.3**
	**Nsp12**	**100**	**96.1**	**96.7**	**98**	**96.7**	**96.7**	**90.8**	**94.8**	**93.5**	**92.8**
**Structural protein**											
**ORF2a**	**GP2**	**100**	**91**	**92.2**	**91.4**	**94.1**	**93.8**	**85.9**	**84.8**	**86.3**	**87.5**
**ORF2b**	**E**	**100**	**93.2**	**93.2**	**95.9**	**95.9**	**93.2**	**95.9**	**89**	**86.3**	**89**
**ORF3**	**GP3**	**99.6**	**93.3**	**92.5**	**79.9**	**93.7**	**94.5**	**81.5**	**80.7**	**80.7**	**81.5**
**ORF4**	**GP4**	**100**	**95.5**	**94.4**	**87.1**	**92.7**	**96.6**	**87.6**	**86**	**86**	**87.1**
**ORF5**	**GP5**	**100**	**90.5**	**92**	**91**	**92**	**92**	**88**	**85.5**	**88**	**85.5**
**ORF6**	**M**	**100**	**95.4**	**96**	**96**	**95.4**	**96**	**94.3**	**93.7**	**92.5**	**92**
**ORF7**	**N**	**100**	**91.9**	**94.3**	**94.3**	**95.1**	**96.7**	**91.9**	**89.4**	**91.1**	**92.7**
